# Development of Synthetic Health Records to Support Urban Planning for Healthy Aging

**DOI:** 10.1093/geroni/igaa057.039

**Published:** 2020-12-16

**Authors:** Yuezhong Liu, Yin Leng Theng

**Affiliations:** Nanyang Technological University, Singapore, Singapore

## Abstract

Urban planning for healthy ageing is about planning for ageing population, which considers the needs of older adults and communities during the planning process and the implications of decisions for human health and well-being. However, access to real electronic health record (EHR) data is hindered by legal, privacy, security, and intellectual property restrictions. The lack of freely distributable health records become one important issue for healthy ageing urban planning. This research develops a source of synthetic health records based on reviewed and meta-analysed evidence on the association between built environmental characteristics related to lifestyle chronic diseases for urban planning. Type 2 Diabetes Mellitus (T2DM) is used as a case study for proof of concept. This research methodology includes three steps: 1) Review and meta-analyse of the individual and built environmental variables related to the prevalence of T2DM. 2) Develop agent-based modelling and simulation for synthetic health records. 3) Evaluate the simulation result with standard healthcare file format in Geographic Information System (GIS) application. The pilot validation compares the annual prevalence of T2DM by age group and ethnicity with the public available health data. The simulation results roughly approximate age, gender and racial group at diagnosis curves (R2 = 0.876), it correctly generated more than 90% of patients for the all age group in Singapore. As a summary, these pilot validated synthetic records could be used as a risk-free (no privacy & security issues) data for supporting urban planning for healthy ageing.

